# Androgen receptors in corticotropin-releasing hormone neurons mediate the sexual dimorphism in restraint-induced thymic atrophy

**DOI:** 10.1073/pnas.2426107122

**Published:** 2025-03-19

**Authors:** Yutong Meng, Yaning Li, Huating Gu, Ziyao Chen, Xiaoyang Cui, Xiaodong Wang

**Affiliations:** ^a^National Institute of Biological Sciences, Beijing and Tsinghua Institute of Multidisciplinary Biomedical Research, Tsinghua University, Beijing 102206, China; ^b^Institute of Biophysics, Chinese Academy of Sciences, Beijing 100101, China; ^c^Zhili College, Tsinghua University, Beijing 100084, China

**Keywords:** androgen receptor, neuroimmunology, sexual dimorphisim, hypothalamic-pituitary-adrenal (HPA) axis, immune suppression

## Abstract

Sexual dimorphism in immune responses is well documented, but the underlying mechanisms remain incompletely understood. Here, we identified a subset of corticotropin-releasing hormone (CRH) neurons that express androgen receptors (ARs) as key mediators of sex differences in restraint-induced immunosuppression. Mechanistically, androgens directly activate AR-positive CRH neurons, enhancing the hypothalamic–pituitary–adrenal axis activation. This results in elevated corticosterone levels in response to restraint stress, leading to increased immune cell apoptosis and immune organ atrophy in male mice. Conditional knockout of ARs in CRH neurons eliminated this sexual dimorphism, highlighting ARs in CRH neurons as pivotal regulators of sex-specific immune responses to stress.

## Significance

Sex differences in immune responses to stress influence disease susceptibility and treatment outcomes, yet the underlying mechanisms are not fully understood. This study identifies androgen receptor (AR)-expressing corticotropin-releasing hormone (CRH) neurons in the mouse hypothalamus as key regulators of sex-specific stress-induced immunosuppression. In male mice, androgen activation of these neurons enhances hypothalamic–pituitary–adrenal axis activity, leading to increased corticosterone levels, thymic atrophy, and immune cell apoptosis. These effects are abolished by specific AR deletion in CRH neurons. By linking androgen signaling in the brain to immune suppression, this study provides critical insights into the neuroendocrine regulation of stress responses.

Traditionally, the nervous and immune systems were thought to function independently. However, it is now well established that neurophysiological states significantly impact immunity and overall health. Indeed, the central nervous system regulates the immune system through various pathways (1–5), with the hypothalamic–pituitary–adrenal (HPA) axis serving as a central mediator of neuroimmune interactions. During stress, corticotropin-releasing hormone (CRH) neurons in the hypothalamic paraventricular nucleus stimulate adrenocorticotrophic hormone (ACTH) release from the pituitary gland, which in turn triggers glucocorticoid release from the adrenal cortex, resulting in broad immunosuppressive effects (6, 7). Restraint stress, a noninvasive psychological stressor, is commonly used as a standard laboratory method to investigate stress-induced immune alterations, manifested by thymic atrophy and thymocyte apoptosis, along with the activation of the HPA axis (8).

Sexual dimorphism in immune response is a widespread phenomenon in mammals, encompassing both innate and adaptive immunity. Females typically exhibit stronger antimicrobial responses than males, resulting in lower infection rates for various bacterial, viral, and parasitic pathogens (9, 10). For example, during the COVID-19 pandemic, males displayed higher infection and mortality rates compared to females (11). In contrast, females are more prone to autoimmune diseases (12), such as systemic lupus erythematosus (SLE), multiple sclerosis (MS), and myasthenia gravis (13). These observations suggest that females exhibit heightened immune reactivity to both self- and non-self-molecular patterns (14, 15).

Research into the mechanisms underlying sexual dimorphism in immune response has primarily focused on two areas: intrinsic differences in immune cell function and external regulatory factors. Female immune cells typically exhibit increased expression of antiviral and proinflammatory genes (16). Conversely, male immune cells, such as B cells, often show reduced functional capacity, including impaired localization to germinal centers (17). Regulatory factors, including sex hormones and environmental influences like nutrition (18) and microbiota composition (19), also play a pivotal role in mediating these differences. Among these factors, androgens are particularly notable as potent immunosuppressors (20–22). For example, androgens negatively regulate the ILC2–DC axis in the skin, thereby diminishing tissue immunity in male mice (23). Studies involving castration further support the immunomodulatory role of androgens, demonstrating that androgen deprivation enhances T cell responses and macrophage activity (24, 25). In this study, we employed physical restraint as a stress model to investigate the stress-induced immunosuppression, specifically focusing on thymic atrophy. Our findings revealed that thymic atrophy, resulting from apoptosis of thymic T cells, showed a significant sex-specific effect. Mechanistic studies identified the androgen receptor presence in corticotropin-releasing hormone (CRH) neurons as a critical mediator of this sexual dimorphism. The presence of the androgen receptor in CRH neurons enhances neuronal activity in response to androgens, leading to elevated glucocorticoid release in male mice via the HPA axis.

## Results

### Restraint-Induced Sexually Dimorphic Immunosuppression.

**Fig. 1.**
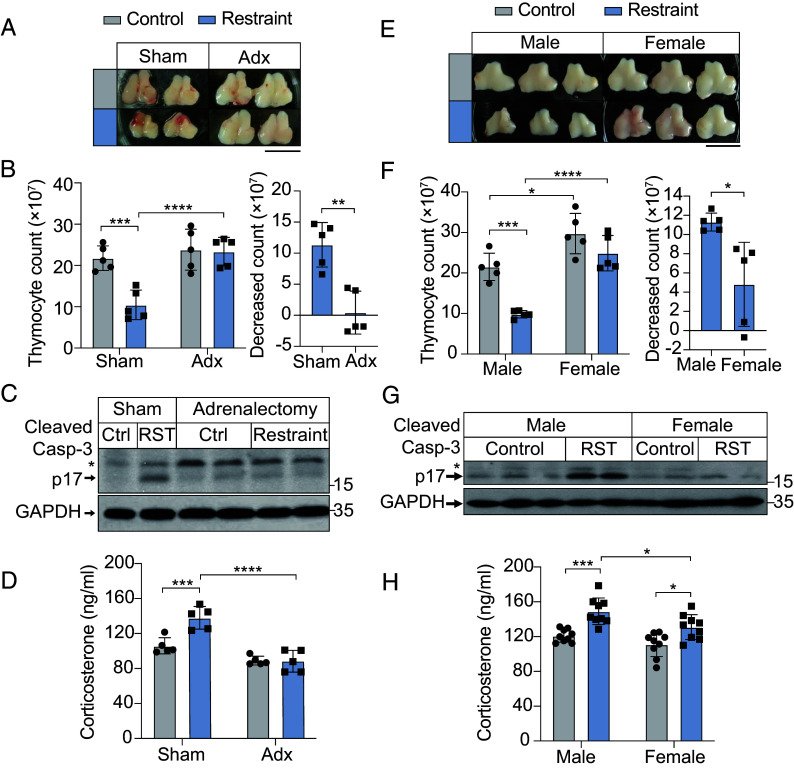
Sexual dimorphism in response to restraint stress. (*A*) Representative images of the thymus size in male mice subjected to sham surgery or adrenalectomy under control or restraint stress conditions. (Scale bar, 1 cm.) (*B*) (*Left*) Thymocyte counts in male mice subjected to sham surgery or adrenalectomy under control or restraint stress conditions. (*Right*) Reduction in thymocyte count, calculated as mean thymocyte count in the Control group minus the thymocyte count for each mouse in the Restraint group. n = 5 mice per group. (*C*) Immunoblot analysis of cleaved caspase-3 (p17) and GAPDH in thymocytes from male mice subjected to sham surgery or adrenalectomy under Ctrl or restraint stress (RST) conditions. * indicates cleaved caspase-3 p19. Each lane represents an individual mouse sample. (*D*) Corticosterone levels in male mice subjected to sham surgery (Sham) or adrenalectomy (Adx) under control or restraint stress conditions. n = 5 mice per group. (*E*) Representative images of thymus size in male and female mice under control (Control) or restraint stress (Restraint) conditions. (Scale bar, 1 cm.) (*F*) (*Left*) Thymocyte counts in male and female mice under control or restraint stress conditions. (*Right*) Decrease in thymocyte counts in male and female mice following restraint stress. n = 5 mice per group. (*G*) Immunoblot analysis of cleaved caspase-3 (p17) and GAPDH in thymocytes from male and female mice under Ctrl or restraint stress (RST) conditions. * indicates cleaved caspase-3 p19. Each lane represents an individual mouse sample. (*H*) Corticosterone levels in male and female mice under control (Control) or restraint stress (Restraint) conditions. n = 9 mice per group. For (*B*, *D*, *F*, and *H*) data are presented as mean ± SD. Statistical analyses were performed using two-way ANOVA (*B* “Thymocyte count,” *D*, *F* “Thymocyte count,” and *H*) and two-tailed unpaired *t* tests (*B* “Decreased count,” *F* “Decreased count”); *P < 0.05, **P < 0.01, ***P < 0.001, and ****P < 0.0001.

To investigate stress-induced immunosuppression, we subjected age-matched mice to acute restraint stress (Restraint, RST) using movement-restricting devices. Untreated littermates served as controls (Control). Restraint stress led to significant thymic atrophy, characterized by reduced thymocyte counts and increased apoptosis, as indicated by elevated levels of cleaved caspase-3 (caspase-3 p17), a marker of apoptosis (26, 27) (Fig. 1 *A*–*C* Sham). Additionally, restraint stress activated the HPA axis, triggering glucocorticoid release, including corticosterone and cortisol (Fig. 1*D* Sham and *SI Appendix*, Fig. S1*A* Sham). Blocking glucocorticoid release through adrenalectomy (Adx) (Fig. 1*D* and *SI Appendix*, Fig. S1*A*) mitigated restraint-induced thymic atrophy, thymocyte loss, and apoptosis (Fig. 1 *A*–*C*). Similarly, the corticosterone synthesis inhibitor Metyrapone prevented thymic atrophy and the reduction in thymocyte count (*SI Appendix*, Fig. S1 *B* and *C*), indicating that restraint-induced immunosuppression relies on glucocorticoid release from the adrenal glands.

To assess whether thymocyte apoptosis, rather than alternative factors such as restraint-induced leukocyte homing (28), underlies restraint-induced thymic atrophy, we treated mice with the caspase inhibitor Ac-DEVD-CHO. This treatment effectively inhibited restraint-induced thymocyte apoptosis (*SI Appendix*, Fig. S1 *D* and *E*) and mitigated both thymic atrophy and the reduction in thymocyte count (*SI Appendix*, Fig. S1 *F* and *G*). These results establish glucocorticoid-induced thymocyte apoptosis as the primary mechanism driving thymic atrophy in response to restraint.

Interestingly, restraint-induced thymic atrophy showed a pronounced sexual dimorphism. Male mice subjected to restraint exhibited more severe thymic atrophy and greater reductions in thymocyte counts than females (Fig. 1 *E* and *F*). Males also exhibited higher levels of the cleaved caspase-3 (Fig. 1*G*) and a larger proportion of Annexin V-positive apoptotic cells (*SI Appendix*, Fig. S1*H*), a marker of apoptosis linked to phosphatidylserine exposure to the outer leaflets of the plasma membrane (29). These findings indicate that the sexual dimorphism in restraint-induced thymic atrophy is attributed to a higher rate of apoptosis in male thymocytes.

To explore why male mice experience greater thymocyte apoptosis, we first tested whether male and female thymocytes differ in glucocorticoid sensitivity. Thymocytes from both sexes cultured with the synthetic glucocorticoid dexamethasone (DEX) showed similar levels of apoptosis (*SI Appendix*, Fig. S1*I*), suggesting that the sex difference arises from disparities in stress hormone levels rather than intrinsic thymocyte sensitivity. Supporting this, male mice exhibited higher corticosterone levels following restraint stress compared to the females (Fig. 1*H*). Similarly, this phenomenon was also observed in the elevation of cortisol, another glucocorticoid in mice (30), following restraint (*SI Appendix*, Fig. S1*J*). This sex difference in stress hormone levels is consistent with human studies showing higher cortisol responses in men under stress (31, 32).

Restraint stress extends its effects beyond the thymus, causing significant atrophy in the spleen and lymph nodes (*SI Appendix*, Fig. S2 *A* and *B*), suggesting a widespread impact on various immune cell populations. Consistently, restraint stress reduced the number of all T cell populations in the thymus (*SI Appendix*, Fig. S2*C*). Flow cytometric analysis revealed that restraint stress reduced the proportions of CD3^+^CD4^−^CD8^−^ double-negative (DN) and CD3^+^CD4^+^CD8^+^ double-positive (DP) T cells while increasing single-positive CD4^+^ (CD4) and CD8^+^ (CD8) T cells (*SI Appendix*, Fig. S2*D*). These results suggest that immature T cells (DN and DP) are more susceptible to restraint stress compared to mature T cells (CD4 and CD8). To determine whether this differential susceptibility is linked to glucocorticoid sensitivity, we sorted these T cell subsets (DN, DP, CD4, CD8) (*SI Appendix*, Fig. S2*H*) and treated them with DEX in vitro. Immature T cells (DN and DP) exhibited higher apoptosis rates (*SI Appendix*, Fig. S2*F*) and greater caspase activation compared to mature T cells (CD4 and CD8) (*SI Appendix*, Fig. S2 *E* and *G*). These findings indicate that restraint-induced immunosuppression primarily affects immature T cells, leading to a significant reduction in immune cell numbers. This selective vulnerability explains the pronounced atrophy observed in the thymus, which predominantly houses immature T cells.

### Regulation of Restraint-Induced Thymic Atrophy by Sex Hormones.

**Fig. 2.**
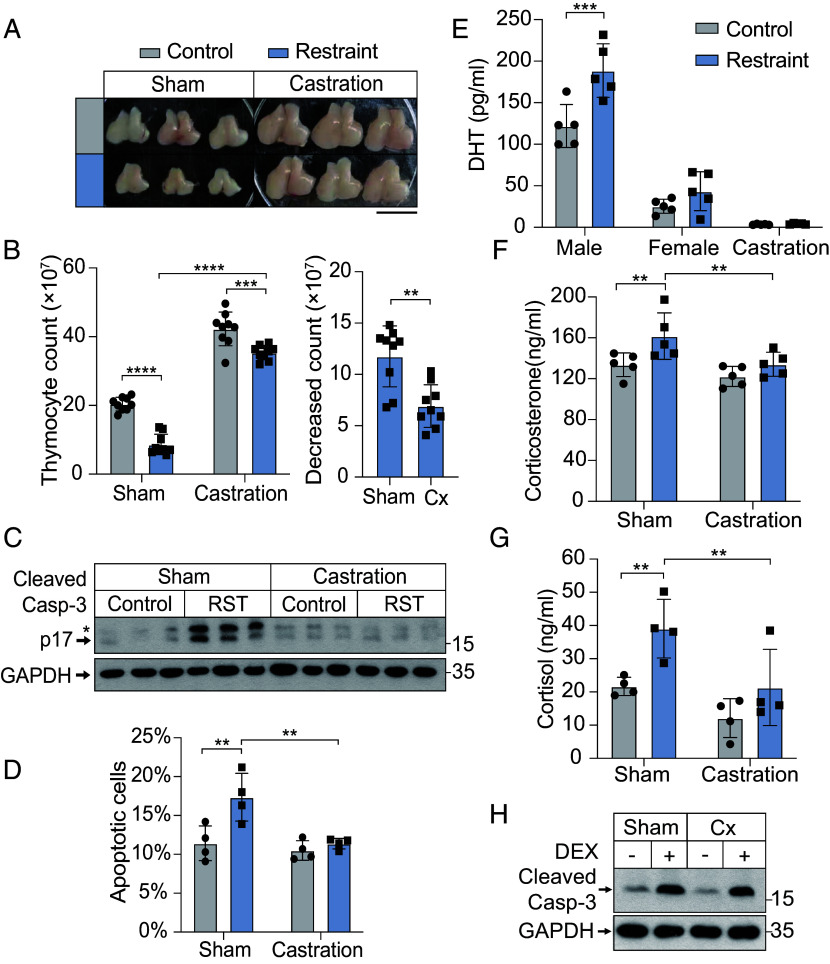
Androgens regulate restraint-induced thymic atrophy. (*A*) Representative images of the thymus size in male mice subjected to sham surgery (Sham) or castration (Cx) under control or restraint stress conditions. (Scale bar, 1 cm.) (*B*) (*Left*) Thymocyte counts in male mice subjected to sham surgery or castration under control or restraint stress conditions. (*Right*) Decrease in thymocyte counts in male mice subjected to sham surgery or castration following restraint stress. n = 9 mice per group. (*C*) Immunoblot analysis of cleaved caspase-3 (p17) and GAPDH in thymocytes from mice subjected to sham surgery or castration under Ctrl or restraint stress (RST) conditions. *indicates cleaved caspase-3 p19. Each lane represents an individual mouse sample. (*D*) Proportions of apoptotic cells in the thymus of male mice subjected to sham surgery or castration under control or restraint stress conditions. n = 4 mice per group. (*E*) DHT levels in male, female, and castrated mice under control or restraint stress conditions. n = 5 mice per group. (*F*) Corticosterone levels in male mice subjected to sham surgery or castration under control or restraint stress conditions. n = 5 mice per group. (*G*) Cortisol levels in male mice subjected to sham surgery or castration under control or restraint stress conditions. n = 4 mice per group. (*H*) Immunoblot analysis of cleaved caspase-3 p17 (Cleaved Casp-3) and GAPDH in thymocytes from male mice subjected to sham surgery or castration, treated with (+) and without (−) 100 nM DEX for 12 h. For (*B, D*–*G*) data are presented as mean ± SD. Statistical analyses were performed using two-way ANOVA (*B* “Thymocyte count,” *D–G*) and two-tailed unpaired *t* tests (*B* “Decreased count”); *P < 0.05, **P < 0.01, ***P < 0.001, and ****P < 0.0001.

In castrated male mice (Cx), where testes were bilaterally removed, the release of dihydrotestosterone (DHT), a major circulating androgen, was effectively abolished during restraint stress (Fig. 2*E*). These castrated mice exhibited markedly reduced thymic atrophy, diminished thymocyte loss, and lower levels of thymocyte apoptosis compared to their littermates who received the sham surgery (Sham) (Fig. 2 *A*–*D*). These findings indicate that androgens play a role in the observed sexual dimorphism in thymic atrophy.

To further explore the role of DHT in mediating sexual dimorphism, we measured stress hormone levels in castrated male mice. Restraint-induced elevations in corticosterone and cortisol were significantly attenuated in castrated mice (Fig. 2 *F* and *G*), indicating that androgens enhance stress hormone release. To confirm that castration did not alter thymocyte sensitivity to glucocorticoids, thymocytes isolated from sham-operated and castrated mice were cultured and treated with DEX. Thymocytes from both groups exhibited similar apoptotic responses to DEX (Fig. 2*H*), demonstrating that the reduced thymic atrophy and apoptosis in castrated males were due to decreased stress hormone levels rather than changes in thymocyte sensitivity.

### Regulation of the HPA Axis by DHT.

**Fig. 3.**
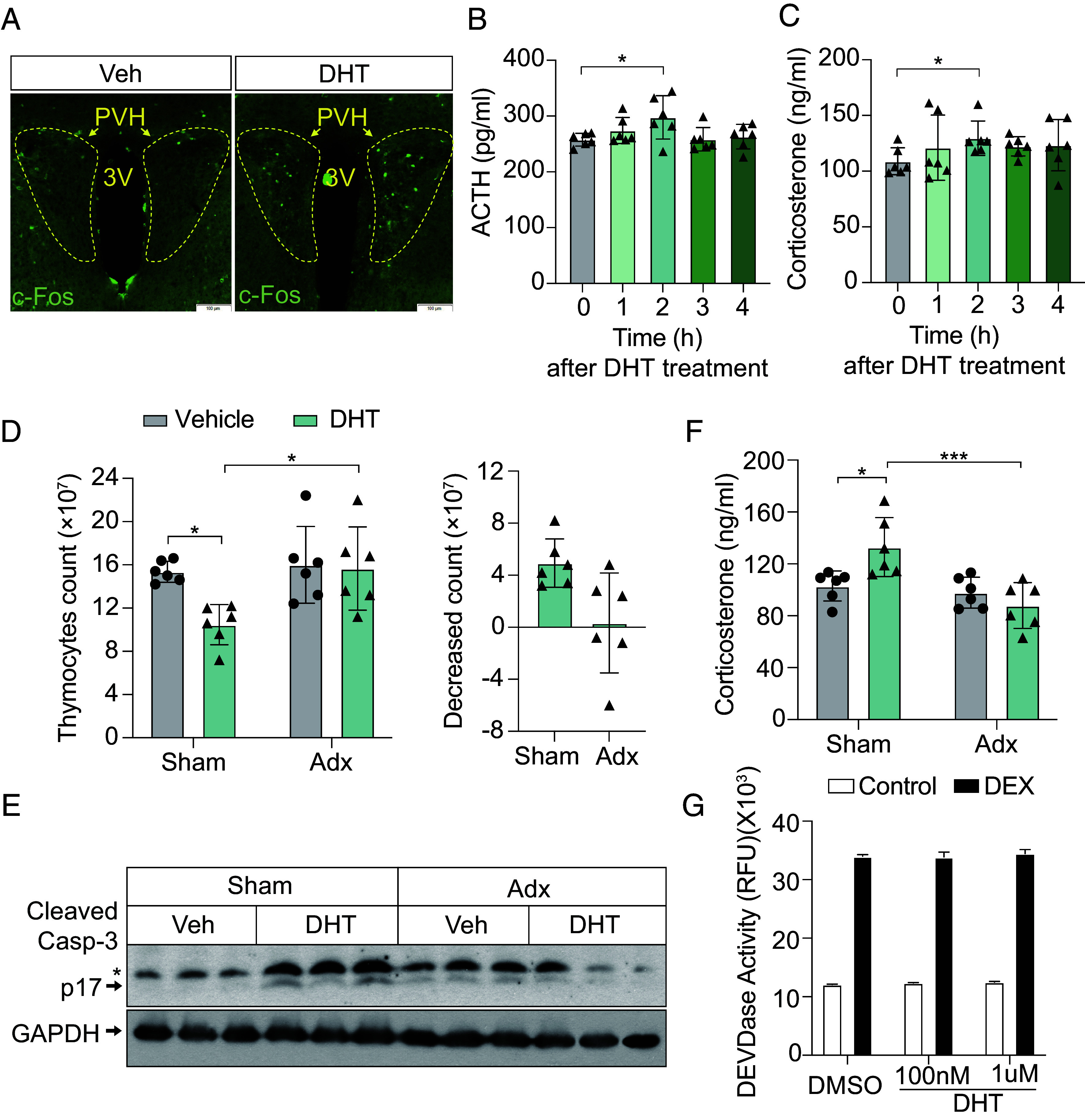
DHT activates the HPA axis. (*A*) Immunofluorescence staining of c-Fos^+^ neurons in the PVH of male mice treated with Veh or DHT. (Scale bar, 100 μm.) (*B*) ACTH levels in male mice following DHT treatment at the indicated time points. n = 6 mice per group. (*C*) Corticosterone levels in male mice following DHT treatment at the indicated time points. n = 6 mice per group. (*D*) (*Left*) Thymocyte counts in male mice that underwent sham surgery (Sham) or adrenalectomy (Adx), followed by treatment with vehicle or DHT. (*Right*) Decreased thymocyte counts in male mice with sham surgery or Adx after DHT treatment. n = 6 mice per group. (*E*) Immunoblot analysis of cleaved caspase-3 (p17) and GAPDH in thymocytes from mice with sham surgery or Adx, treated with vehicle or DHT. *indicates cleaved caspase-3 p19. Each lane represents a sample from an individual mouse. (*F*) Corticosterone levels in male mice subjected to sham surgery or adrenalectomy and treated with vehicle and DHT. n = 6 mice per group. (*G*) DEVDase activity in thymocytes treated with 100 nM DEX in the absence (DMSO) or presence of different concentrations of DHT for 10 h. For (*B* and *C*) data are presented as mean ± SEM. For (*D*, *F*, and *G*) data are presented as mean ± SD. Statistical analyses were performed using one-way ANOVA (*B* and *C*), two-way ANOVA (*D* “Thymocyte count,” *F*), and two-tailed unpaired *t* tests (*D* “Decreased count”); *P < 0.05, **P < 0.01, and ***P < 0.001.

To investigate how androgens enhance glucocorticoid release, we administered DHT intraperitoneally to male mice and examined the paraventricular nucleus of the hypothalamus (PVH), a critical regulator of stress hormone release through the HPA axis (33–35). Compared to the control group treated with vehicle (Veh), DHT treatment activated PVH neurons, evidenced by increased c-Fos expression, a marker of neuronal activation (Fig. 3*A*). Among these PVH neurons, CRH-producing neurons play a central role in driving ACTH release from the anterior pituitary, which subsequently stimulates corticosterone release from the adrenal glands (36–38). Consistent with the activation of PVH neurons, DHT administration elevated ACTH and corticosterone levels (Fig. 3 *B* and *C*) and caused thymocyte loss and apoptosis (Fig. 3 *D* and *E* Sham).

To confirm the necessity of HPA axis activation for DHT-induced thymic atrophy, male mice underwent adrenalectomy before DHT treatment. In adrenalectomized mice, DHT failed to induce corticosterone release (Fig. 3*F*) or trigger thymocyte loss by apoptosis (Fig. 3 *D* and *E*), indicating that DHT-induced thymic atrophy depends on HPA axis activation. Moreover, in vitro treatment of thymocytes with DHT or DEX demonstrated that DHT alone did not induce apoptosis nor alter DEX-induced apoptosis (Fig. 3*G*). These results indicate that DHT promotes restraint stress-induced thymic atrophy by enhancing glucocorticoid release through HPA axis activation, rather than directly influencing glucocorticoid-mediated thymocyte apoptosis.

Given that testes also produce small amounts of estrogen, we examined whether estradiol (E2) could influence stress hormone release. Unlike DHT, E2 treatment did not activate PVH neurons (*SI Appendix*, Fig. S3*A*) or induce corticosterone or cortisol release (*SI Appendix*, Fig. S3 *B* and *C*).

Since C1 neurons in the medulla oblongata are known to mediate PVH activation in response to various physical stressors, including restraint (39–41), we examined whether DHT activates PVH via C1 neurons. While restraint robustly activated C1 neurons, DHT treatment did not (*SI Appendix*, Fig. S3*D*). These findings suggest that DHT directly activates PVH neurons, bypassing the involvement of upstream C1 neurons.

### Activation of CRH Neurons by DHT.

The PVH contains various types of neurons, of which CRH neurons play a key role in activating the HPA axis (42). To determine whether DHT directly activates CRH-positive neurons in the PVH, male mice were treated with DHT, and costaining for CRH and c-Fos was performed. Indeed, it was the CRH neurons that were activated by DHT treatment, as evidenced by the colocalization of CRH and c-Fos signals (*SI Appendix*, Fig. S4*A*).

**Fig. 4.**
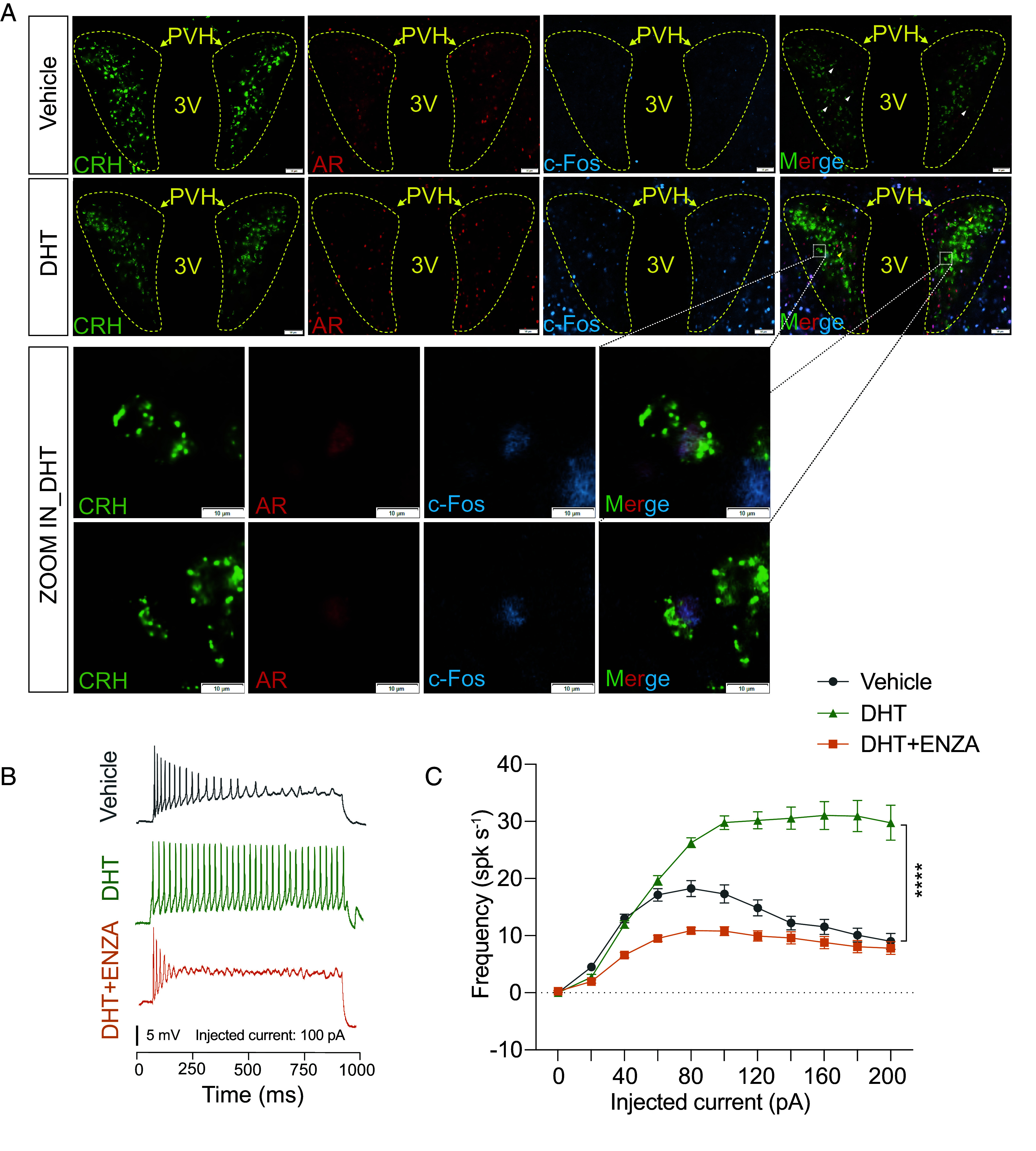
DHT activates AR-positive CRH neurons. (*A*) Immunofluorescence staining of AR and c-Fos in the PVH of *Crh^cre+/+^*; *Ai6^+/+^*male mice treated with vehicle or DHT. White arrows indicate the colocalization of CRH and AR in the vehicle group, while yellow arrows indicate the colocalization of CRH, AR, and c-Fos in the DHT group. (Scale bar, 50 μm.) The panels below show magnified views of the region within the white dashed box in the DHT group. (Scale bar, 10 μm.) (*B*) Representative traces of depolarization-induced spiking activity in CRH neurons from male mice treated with Vehicle, DHT, or DHT combined with the AR antagonist ENZA. Injected current = 100 pA. (*C*) Quantitative analysis of the firing rates in CRH neurons in response to step depolarizing current injections. For (*C*) data are presented as mean ± SEM. Statistical analysis was performed using two-way ANOVA (*C*); *P < 0.05, **P < 0.01, ***P < 0.001, and ****P < 0.0001.

To further investigate the mechanism by which DHT activates these CRH neurons, we generated *Crh^cre+/+^*; *Ai6^+/+^* mice, in which CRH-positive neurons are labeled with the enhanced green fluorescent protein variant ZsGreen1. Immunofluorescence staining of the PVH revealed the presence of androgen receptors (ARs) in the CRH neurons (*SI Appendix*, Fig. S4*B*). While previous studies have reported AR expression in the PVH of rats and mice (43, 44), its precise function there has remained unclear. Here, we observed the colocalization of AR, CRH, and c-Fos in PVH neurons following DHT treatment (Fig. 4*A* and Movie S1), indicating that DHT may directly bind to ARs in CRH neurons, leading to their activation.

To investigate whether DHT directly enhances the excitability of CRH neurons, we conducted ex vivo patch-clamp recordings on CRH neurons in the PVH. DHT treatment significantly increased the firing rates of CRH neurons in response to step depolarizing current injections compared to vehicle-treated controls (Fig. 4 *B*, *Top* and *Middle*, Fig. 4*C*). Additionally, this DHT-induced excitation was abolished by pretreatment with the AR antagonist enzalutamide (ENZA) (Fig. 4 *B*, *Bottom*, Fig. 4*C*). These findings demonstrated that DHT directly enhanced the excitability of CRH neurons via AR-dependent mechanisms.

### AR-Positive CRH Neurons Regulate Sexual Dimorphism.

To further investigate the specific role of ARs in CRH neurons, we conditionally knocked out ARs in these neurons. To this end, male offspring of a cross between *AR^flox+/y^* male mice and *AR^flox+/+^*; *Crh^cre+/−^* female mice were generated. Among these, half were *AR^flox+/y^*; *Crh^cre+/−^* [androgen receptor conditional knockout (AR cKO)], with ARs selectively deleted in CRH neurons, while the other half were *AR^flox+/y^*(wild type, WT) and served as controls. Genotyping was conducted before experiments to ensure accuracy, and littermates were used to minimize environmental and age-related variables.

**Fig. 5.**
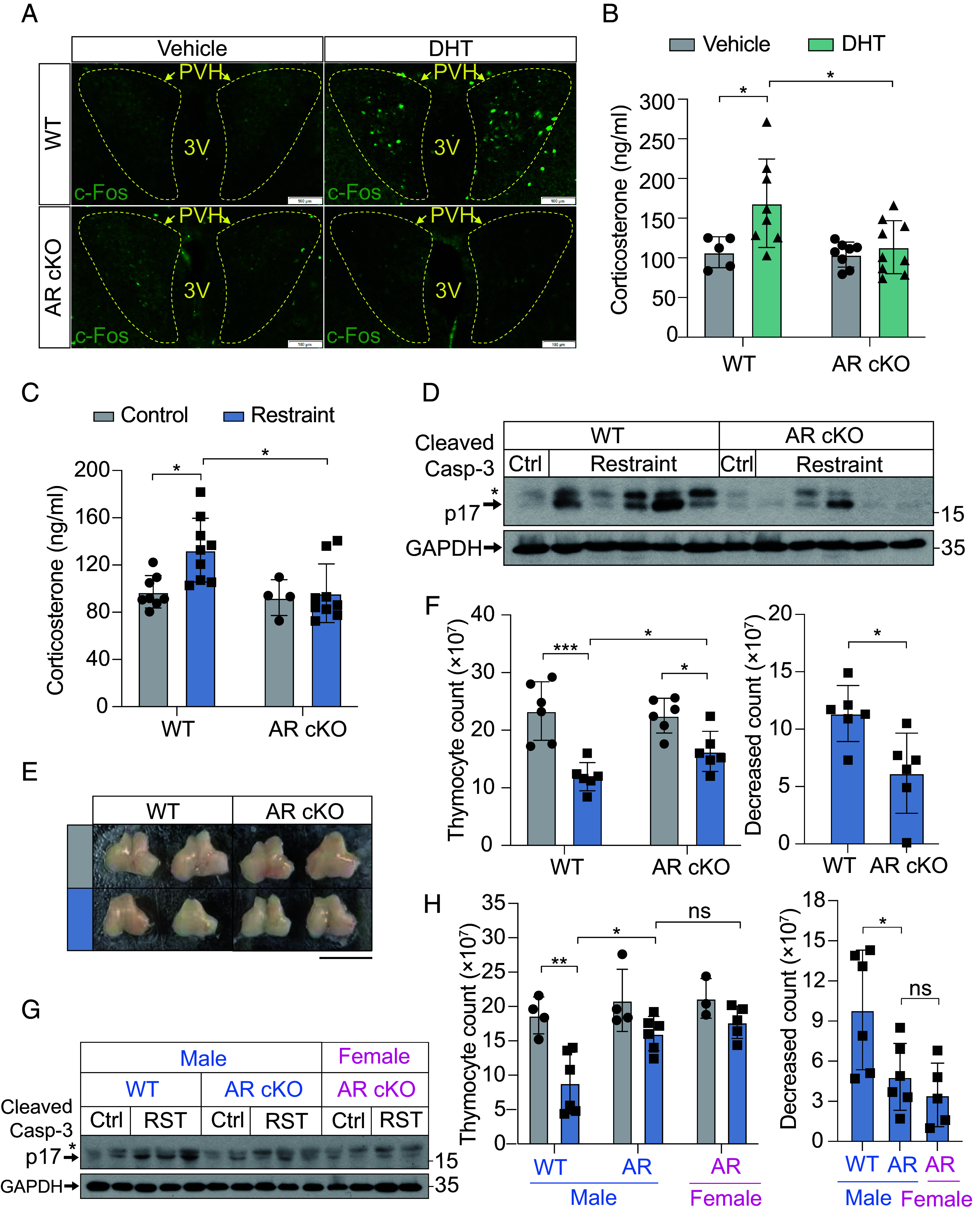
DHT activates CRH neurons in an androgen receptor–dependent manner. (*A*) Immunofluorescence staining of c-Fos^+^ neurons in the PVH from WT male mice and AR cKO male mice treated with vehicle or DHT. (Scale bar, 100 μm.) (*B*) Corticosterone levels in WT and AR cKO male mice treated with vehicle or DHT. n = 5 mice (WT Vehicle), n = 8 mice (WT DHT and AR cKO Vehicle), n = 9 mice (AR cKO DHT). (*C*) Corticosterone levels in WT and AR cKO male mice under control or restraint stress (Restraint) conditions. n = 8 mice (WT Control), n = 4 mice (AR cKO Control), n = 9 mice (Restraint groups). (*D*) Immunoblot analysis of cleaved caspase-3 (p17) and GAPDH in thymocytes from WT and AR cKO male mice under Ctrl or restraint stress (Restraint) conditions. *indicates cleaved caspase-3 p19. Each lane represents a sample from an individual mouse. (*E*) Representative images of the thymus size in WT and AR cKO male mice under control or restraint stress conditions. (Scale bar, 1 cm.) (*F*) (*Left*) Thymocyte counts in WT and AR cKO male mice under control or restraint stress conditions. (*Right*) Decrease in thymocyte counts in WT and AR cKO male mice following restraint stress. n = 6 mice per group. (*G*) Immunoblot analysis of cleaved caspase-3 (p17) and GAPDH in thymocytes from WT male, AR cKO male, and AR cKO female mice under control or restraint stress (RST) conditions. *indicates cleaved caspase-3 p19. Each lane represents a sample from an individual mouse. (*H*) (*Left*) Thymocyte counts in WT male, AR cKO male, and AR cKO female mice under control or restraint stress conditions. (*Right*) Decrease in thymocyte counts following restraint stress. n = 4 mice (WT male control and AR male control), n = 6 mice (WT male restraint and AR male restraint), n = 3 mice (AR female control), n = 5 (AR female restraint). For (*B*, *C*, *F*, and *H*) data are presented as mean ± SD. Statistical analyses were performed using two-way ANOVA (*B*, *C*, and *F* “Thymocyte count” and *H*), and two-tailed unpaired *t* tests (*F* “Decreased count”); *P < 0.05, **P < 0.01, and ***P < 0.001.

To determine whether AR knockout in CRH neurons disrupts DHT-induced HPA axis activation, we administered DHT to WT and AR cKO mice. In WT mice, DHT treatment activated PVH neurons as measured by c-Fos staining, whereas no such staining was detected in AR cKO mice (Fig. 5*A* and *SI Appendix*, Fig. S5*A*). Consistently, DHT-induced corticosterone release observed in WT mice was abolished in AR cKO mice (Fig. 5*B*). These results confirm that ARs in CRH neurons are essential for DHT-mediated HPA axis activation.

When subjected to restraint stress, AR cKO mice showed significantly attenuated corticosterone release compared to WT mice (Fig. 5*C*). This reduction in corticosterone was accompanied by decreased thymocyte apoptosis in AR cKO mice (Fig. 5*D*). Additionally, the thymic atrophy and reduction in thymocyte count observed in WT mice were reduced in AR cKO mice (Fig. 5 *E* and *F*), indicating that AR-positive CRH neurons amplify restraint-induced immunosuppression. Notably, conditional knockout of AR in CRH neurons abolished the sexual dimorphism in restraint-induced thymocyte apoptosis and thymic atrophy (Fig. 5 *G* and *H* and *SI Appendix*, Fig. S5*B*). These findings demonstrate that AR-positive CRH neurons, activated by DHT during restraint stress, are key mediators of the sexual dimorphism in thymic responses to stress.

## Discussion

This study highlights the sexual dimorphism observed in restraint-induced immunosuppression, with male mice exhibiting more pronounced immune cell apoptosis. This difference is regulated by AR-positive CRH neurons in the PVH. During restraint stress, male mice secrete more androgens, which bind directly to ARs in CRH neurons, increasing their excitability. This leads to a more robust activation of the HPA axis in male mice compared to females, resulting in higher levels of stress hormones. Among these, glucocorticoids, a class of stress hormones, are well known to induce immune cell apoptosis (45, 46). Elevated glucocorticoid levels in male mice contribute to greater immune cell apoptosis and immune organ atrophy.

The nervous system regulates the immune system through various complex mechanisms. One pathway involves direct neuroimmune interactions through the innervation of immune organs. For instance, when stimulated by the vagus nerve, the sympathetic nerves in the spleen release norepinephrine, prompting a specialized subset of T cells to secrete acetylcholine, which modulates macrophage function (3, 47, 48). Another pathway involves indirect regulation, such as the activation of the HPA axis, which promotes glucocorticoid release, inducing immune cell apoptosis and influencing immune processes like immune cell migration (28). Restraint stress is a classic model for studying the effects of HPA axis activation on the immune system (8). This study complements existing research on restraint-induced immunosuppression, demonstrating that different T cell lineages exhibit varying sensitivity to glucocorticoids, with immature T cells being the most susceptible. This finding explains why thymic atrophy is more severe than defects in other immune organs.

This study also identifies significant sex differences in the immune response to restraint stress, primarily due to stronger HPA axis activation in male mice. Hormonal differences in stress response have been reported in both mice and humans (31, 32, 49, 50). Sexual dimorphism in immune responses is well documented, with females generally exhibiting stronger immune activity. This is reflected in the prevalence of autoimmune diseases, infection outcomes, and response to vaccinations. However, the mechanisms underlying immune sexual dimorphism remain incompletely understood. While sex hormones such as estrogens and androgens have been shown to directly regulate immune cell functions and contribute to sexual dimorphism (17, 51), this study highlights the androgenic regulation of the HPA axis through AR-positive CRH neurons. This pathway is a crucial determinant of male susceptibility to stress-induced immune modulation, offering a perspective on sexual dimorphism in stress-related immune responses. However, other studies have reported that in rats, females exhibit stronger HPA axis activity and release more stress hormones (52). This discrepancy may stem from the higher expression of ARs in the PVH of rats compared to mice (43, 44). Consequently, DHT may activate these neurons more strongly in rats, leading to inhibitory effects via negative feedback mechanisms. Therefore, the regulation of the HPA axis by DHT is a complex and dynamic process, influenced by factors such as AR expression levels, treatment duration, and other variables. Certain diseases can induce immunosuppression through HPA axis activation and stress hormone release. For instance, stroke patients frequently develop stroke-associated pneumonia (SAP), partly driven by HPA axis activation (53, 54). SAP is more common in males (55) and can be influenced by androgens (56), suggesting that DHT-regulated HPA axis activity may contribute to sex differences in certain diseases.

## Materials and Methods

### Animals.

All animal experiments adhered to the Chinese Ministry of Health’s national guidelines for laboratory animal housing and care. Additionally, they were reviewed, approved, and conducted in accordance with the Institutional Animal Care and Use Committee of the National Institute of Biological Sciences, Beijing. Mice were housed in groups of five per cage under specific-pathogen-free conditions, maintained on a 12-h light/dark cycle at 22 to 25 °C, with food and water available ad libitum. Wild-type (C57BL/6J), Ai6, CRH-Cre, and AR-flox mice were used in this study. B6.Cg-*Gt(ROSA)26Sor^tm6(CAG-ZsGreen1)Hze^*/J (Ai6) (Strain #:007906) were purchased from The Jackson Laboratory. B6(Cg)-*Crh^tm1(cre)Zjh^*/J (CRH-Cre) (Strain #:012704) were kindly provided by Minmin Luo at the National Institute of Biological Sciences (NIBS), purchased from The Jackson Laboratory. Ar-flox mice (Strain NO. T052312) were purchased from GemPharmatech (Nanjing, China). Genotyping for each strain was conducted according to the guidelines provided on the Jackson Laboratory website and the GemPharmatech (Nanjing, China) website. Relevant mice were crossed to generate *AR^flox+/+^*; *Crh^cre+/−^* (female), *AR^flox+^*; *Crh^cre+/−^* (male) plus respective *AR^flox+/+^* (female), *AR^flox+^* (male) controls. *Crh^cre+/+^*; *Ai6^+/+^* mice were generated as reporter mice. One week before the restraint and DHT treatment experiments, as well as after surgery, mice were housed individually. All experiments were conducted on 6 to 8-wk-old mice with age- and sex-matched controls.

### In Vivo Interventions and Procedures.

#### Restraint stress model.

Mice were individually housed and acclimated to the environment before undergoing stress interventions. Restraint stress was induced by placing the mice into well-ventilated restraint cylinders (or cuboids) without squeezing. The duration and frequency of stress exposure varied as indicated. To detect thymic atrophy, thymocyte apoptosis, and neuron activation, restraint was performed for 12 h (overnight, from Day 0 22:00 to Day 1 10:00). To detect hormone levels (corticosterone, cortisol, and DHT), restraint was performed for 1 h (from Day 1 11:00 to 12:00). Both are single-time acute stress models, but samples cannot be collected simultaneously due to differing timelines for peak events. Hormone levels reach their peak at 1 h poststimulus and then begin to decline, while cell death occurs later, requiring more time after hormone release to become detectable. Therefore, the optimal time for assessing cell death is at 12 h, when the effects are most pronounced. Given that the mice’s hormones fluctuate periodically throughout the day, the start and end times of each experiment were controlled to be at the same time of day.

#### DHT injections.

DHT was purchased from Solarbio (ID0310). DHT was dissolved with DMSO to prepare a 100 mM stock solution. The working solution was diluted with Sulfobutylether-β-Cyclodextrin (SBE-β-CD) before the experiment. SBE-β-CD was purchased from MedChemExpress (HY-17031) and dissolved to 20% (w/v) in saline as a working solution. Each 6-wk-old male mouse was intraperitoneally injected with 100 µL of a 5 mM DHT solution (7.3 mg/kg). Each mouse was injected twice (Day 0 22:00 and Day 1 10:00). Samples were collected 2 h after the last injection (Day 1 12:00). The mice in control groups were injected with the same volume of vehicle (DMSO in SBE-β-CD) at the same time.

#### E2 injections.

E2 was purchased from MedChemExpress (HY-B0141). E2 was dissolved with DMSO to prepare a 100 mM stock solution. The working solution was diluted with 20% SBE-β-CD before the experiment. Each 6-wk-old mouse was intraperitoneally injected with 100 µL of a 5 mM E2 solution (8.8 mg/kg). Each mouse was injected twice (Day 0 22:00 and Day 1 10:00). Samples were collected 2 h after the last injection (Day 1 12:00). The mice in control groups were injected with the same volume of vehicle (DMSO in SBE-β-CD) at the same time.

#### Ac-DEVD-CHO injections.

Ac-DEVD-CHO was purchased from Selleckchem (S7901). Ac-DEVD-CHO was dissolved with DMSO and diluted with 20% SBE-β-CD. Before restraint stress, mice were intraperitoneally injected with vehicle (DMSO in SBE-β-CD) or Ac-DEVD-CHO (3 mg/kg).

#### ENZA injections.

ENZA was purchased from MedChemExpress (HY-70002). ENZA was dissolved with DMSO to prepare a 100 mM stock solution. The working solution was diluted with Sulfobutylether-β-Cyclodextri (SBE-β-CD) before the experiment. SBE-β-CD was purchased from MedChemExpress (HY-17031) and dissolved to 20% (w/v) in saline as a working solution. Each 6-wk-old male mouse was intraperitoneally injected with 100 µL of a 5 mM ENZA solution (11 mg/kg) before DHT treatment. Each mouse was injected twice (Day 0 22:00 and Day 1 10:00). Samples were collected 2 h after the last injection (Day 1 12:00). The mice in control groups were injected with the same volume of vehicle (DMSO in SBE-β-CD) at the same time.

#### Metyrapone injections.

Metyrapone was purchased from MedChemExpress (HY-B1232). Metyrapone was dissolved with DMSO and diluted with 20% SBE-β-CD. Before restraint stress, mice were intraperitoneally injected with vehicle (DMSO in SBE-β-CD) or Metyrapone (50 mg/kg).

#### CRH neuronal recording.

Brain slices containing the PVH were obtained from *Crh^cre+/+^*; *Ai6^+/+^* male mice. Mice were treated with Vehicle, DHT, or ENZA. Brains were rapidly extracted and placed in an ice-cold oxygenated cutting solution (95% O_2_, 5% CO_2_, 228 mM sucrose, 2.5 mM KCl, 26 mM NaHCO_3_, 11 mM glucose, 7 mM MgSO_4_, 1 mM NaH_2_PO_4_, and 0.5 mM CaCl_2_). Coronal brain slices (400 μm) were sectioned using a vibratome (VT 1200S, Leica Microsystems, Wetzlar, Germany). Slices were incubated at 28 °C in oxygenated artificial cerebrospinal fluid (ACSF: 2.5 mM KCl, 119 mM NaCl, 1 mM NaH_2_PO_4_, 10 mM glucose, 26 mM NaHCO_3_, 1.3 mM MgSO_4_, and 2.5 mM CaCl_2_) for 30 min, then maintained at room temperature for 1 h before transfer to the recording chamber, where ACSF was perfused at 1 mL/min. PVH slices were visualized using a 40× Olympus water immersion lens with differential interference contrast optics (DIC, Olympus Inc., Japan), and a CCD camera (Q- Imaging Rolera-XR, BC, Canada). Patch pipettes were made by borosilicate glass capillary tubes (Cat #64-0793, Warner Instruments, Hamden, CT) using a pipette puller (Narishige Inc., Tokyo, Japan). For current-clamp recordings of action potentials, pipettes were filled with solution (135 M-methanesulfonate, 10 mM HEPES, 1 mM EGTA, 1 mM Na-GTP, 4 mM Mg-ATP, and 2% neurobiotin, pH 7.4). After establishing whole-cell configuration and equilibration of the pipette solution with the cytoplasm, series resistance was compensated to 10 to 15 MΩ. Recordings with series resistances of >15 MΩ were excluded. Depolarizing currents (20 to 200 pA, in 20 pA increments) were injected. Pipette resistance ranged from 4 to 6 MΩ. Current and voltage signals were recorded with MultiClamp 700B and Clampex 10 software (Molecular Devices).

### Surgical Interventions.

#### Adrenalectomy.

Mice were anesthetized with Avertin (intraperitoneal; 20 mg/mL working solution; 0.2 mL per 10 g body weight; working solution prepared with 2,2,2-tribromomethanol and tert-amyl alcohol). Bilateral adrenalectomy was performed via dorsolateral subcostal incisions. Sham-operated mice served as controls. Adrenalectomized mice were allowed to recover for at least 7 d before further procedures.

#### Castration.

Mice were anesthetized with Avertin. A bilateral orchiectomy was performed through a small incision. Sham-operated mice served as controls. Mice were allowed to recover for at least 7 d before further procedures.

### In Vitro Procedures.

#### Thymocyte counting.

Mice were killed and perfused with PBS. The thymus was then harvested and ground using a 40 μm cell strainer to obtain a thymocyte suspension. The number of thymocytes was counted using a hemocytometer.

#### Thymocyte primary culture.

Thymocytes were maintained in RPMI-1640 medium (Gibco) supplemented with 10% fetal bovine serum (FBS, Gibco) and 1% penicillin/streptomycin (Pen/Strep, Gibco). Cells were incubated at 37 °C in a humidified atmosphere with 5% CO_2_.

#### Reagents.

The concentrations of each of the reagents were as follows: DEX (Selleckchem S4028, 100 nM) and DHT (Solarbio ID0310, 100 nM and 1 μM).

#### Flow cytometry.

Single-cell suspensions of thymocytes were prepared and incubated in ice-cold MACS buffer (PBS with 1 mM EDTA and 2% FBS) containing 10 μg/mL CD16/CD32 antibody (553142, Clone 2.4G2, BD PharMingen) for 30 min at 4 °C. Afterward, cells were stained with the following antibodies: anti-CD45 (557235, Clone 30-F11, BD PharMingen), anti-CD3e (553062, Clone 145-2C11, BD PharMingen), anti-CD4 (553051, Clone RM4-5, BD PharMingen), and anti-CD8 (563898, Clone 53-6.7, BD Horizon). Data were collected using a BD FACSAria Fusion flow cytometer and analyzed with FlowJo software (TreeStar). The gating strategy was as follows: DN T cell (CD45^+^CD4^−^CD8^−^), DP T cell (CD45^+^CD4^+^CD8^+^), CD4^+^ T cell (CD45^+^CD4^+^CD8^−^), and CD8^+^ T cell (CD45^+^CD4^−^CD8^+^).

#### Apoptotic cell detection.

Apoptotic cell detection was performed using the Annexin V-FITC Apoptosis Detection Kit (Beyotime, C1062S) according to the manufacturer’s instructions. Briefly, cells were incubated with the binding buffer containing Annexin V-FITC for 20 min at room temperature. The FITC-positive cells were then analyzed using a BD FACSAria Fusion flow cytometer.

#### DEVDase activity detection.

The DEVDase activity detection was performed using the Caspase-Glo® 3/7 Assay System (Promega, G8091). Briefly, thymocytes were plated in 96-well culture plates. After the DEX or DHT treatment, Caspase-Glo® 3/7 Reagent was added to each well. The reaction was incubated on a horizontal shaker at room temperature for 30 min. Luminescent signals were then measured using a plate reader (PerkinElmer, EnSpire).

#### Activated caspase-3 detection.

The activity of caspase-3 detection was performed using the BD Pharmingen™ PE Active Caspase-3 Apoptosis Kit (BD, 550914). Briefly, cells were incubated in BD Cytofix/Cytoperm™ solution for 20 min on ice. The cells were then washed and resuspended in the BD Perm/Wash™ buffer (1×) and incubated with the antibody for 30 min at room temperature. The cells with activated caspase-3 were subsequently analyzed by flow cytometry.

### ELISA.

#### Corticosterone ELISA.

Serum corticosterone levels were quantified using the QuicKey Pro Mouse CORT(Corticosterone) ELISA Kit (Elabscience, E-OSEL-M0001) following the manufacturer’s instructions.

#### Cortisol ELISA.

Serum cortisol levels were quantified using the General Cortisol ELISA Kit (Cor) (ABclonal, RK00639) following the manufacturer’s instructions.

#### ACTH ELISA.

Serum ACTH levels were quantified using a commercial ACTH ELISA kit (Elabscience, E-EL-M0079c) following the manufacturer’s instructions.

#### DHT ELISA.

Serum DHT levels were quantified using the DHT ELISA Kit (Elabscience, E-EL-0031) following the manufacturer’s instructions.

#### CRH ELISA.

Serum CRH levels were quantified using the CRH ELISA Kit (Elabscience, E-EL-M0351) following the manufacturer’s instructions.

#### Immunofluorescence and antibodies.

For tissue section preparation, mice were deeply anesthetized and underwent intracardial perfusion with PBS followed by 4% paraformaldehyde (PFA). Brains were then fixed in 4% PFA overnight at 4 °C and dehydrated in 30% sucrose for cryoprotection. Brain slices with a thickness of 20 to 30 μm were cut using a cryostat (Leica). Slides were blocked and permeabilized with SuperBlock blocking buffer (Thermo Scientific) supplemented with 0.1% Triton X-100 for 1 h at room temperature. They were then incubated with primary antibodies in phosphate-buffered saline with Tween-20 (PBST) buffer in a humidified chamber at 4 °C overnight. Primary antibodies include c-Fos (Abcam, Rabbit ab190298), c-Fos (Abcam, Sheep ab6167), c-Fos (Abcam, Mouse ab208942), Androgen Receptor (ABclonal, A19611), and CRH/CRF (Proteintech, 10944-1-AP). After primary antibody incubation, slices were washed three times with PBST and incubated with fluorochrome-conjugated secondary antibodies at room temperature for 1 to 2 h in the dark. Secondary antibodies included donkey anti-rabbit Alexa Fluor™ 488 (Invitrogen), donkey anti-rabbit Alexa Fluor™ 555 (Invitrogen), donkey anti-mouse Alexa Fluor Fluor™ 555 (Invitrogen), donkey anti-rabbit Alexa Fluor™ 647 (Invitrogen), and donkey anti-sheep Alexa Fluor™ 647 (Invitrogen). After three washes with PBST, slices were mounted with 50% glycerol containing DAPI. Images were obtained using an Olympus VS120 or Olympus VS200. 3D images were obtained using Nikon AX.

#### Western Blot and antibodies.

Western blot samples were prepared by lysing total thymocytes in 1x SDS loading buffer. The following primary antibodies were used: cleaved caspase-3 [Cell Signaling Technology (CST), #9664], GAPDH (MBL, M171-3), and caspase-9 (CST, #9508).

### Statistics.

Statistical analyses were performed using GraphPad Prism 10. For comparisons between two groups, unpaired two-tailed Student’s *t* tests were applied. For multiple comparisons, one-way ANOVA followed by Dunnett’s multiple-comparisons test and two-way ANOVA followed by Sidak’s multiple-comparisons test were used. *P* < 0.05 was considered statistically significant; *P < 0.05, **P < 0.01, ***P < 0.001, and ****P < 0.0001.

## Supplementary Material

Appendix 01 (PDF)

Movie S1. 3D visualization of AR, c-Fos, and CRH in the PVH region of *Crhcre^+/+^;Ai6^+/+^* male mice following DHT treatment.

## Data Availability

All study data are included in the article and/or supporting information.
