# Supplementary material for: Androgen receptors in corticotropin-releasing hormone neurons mediate the sexual dimorphism in restraint-induced thymic atrophy
Source: Proc Natl Acad Sci U S A. 2025 Mar 19;122(12):e2426107122. doi: 10.1073/pnas.2426107122 (PMC11962470; doi:10.1073/pnas.2426107122)
Supplement: Supplementary file 1 — Appendix 01 (PDF) [file pnas.2426107122.sapp.pdf]

PNAS

Supporting Information for

Androgen Receptors in Corticotropin-Releasing Hormone Neurons Mediate the Sexual Dimorphism in Restraint-Induced Thymic Atrophy

Yutong Meng, Yaning Li, Huating Gu, Ziyao Chen, Xiaoyang Cui, Xiaodong Wang

Corresponding author: Xiaodong Wang
Email: wangxiaodong@nibs.ac.cn

This PDF file includes:

Figures S1 to S5
Legend for Movie S1

Other supporting materials for this manuscript include the following:

Movies S1

Fig. S1

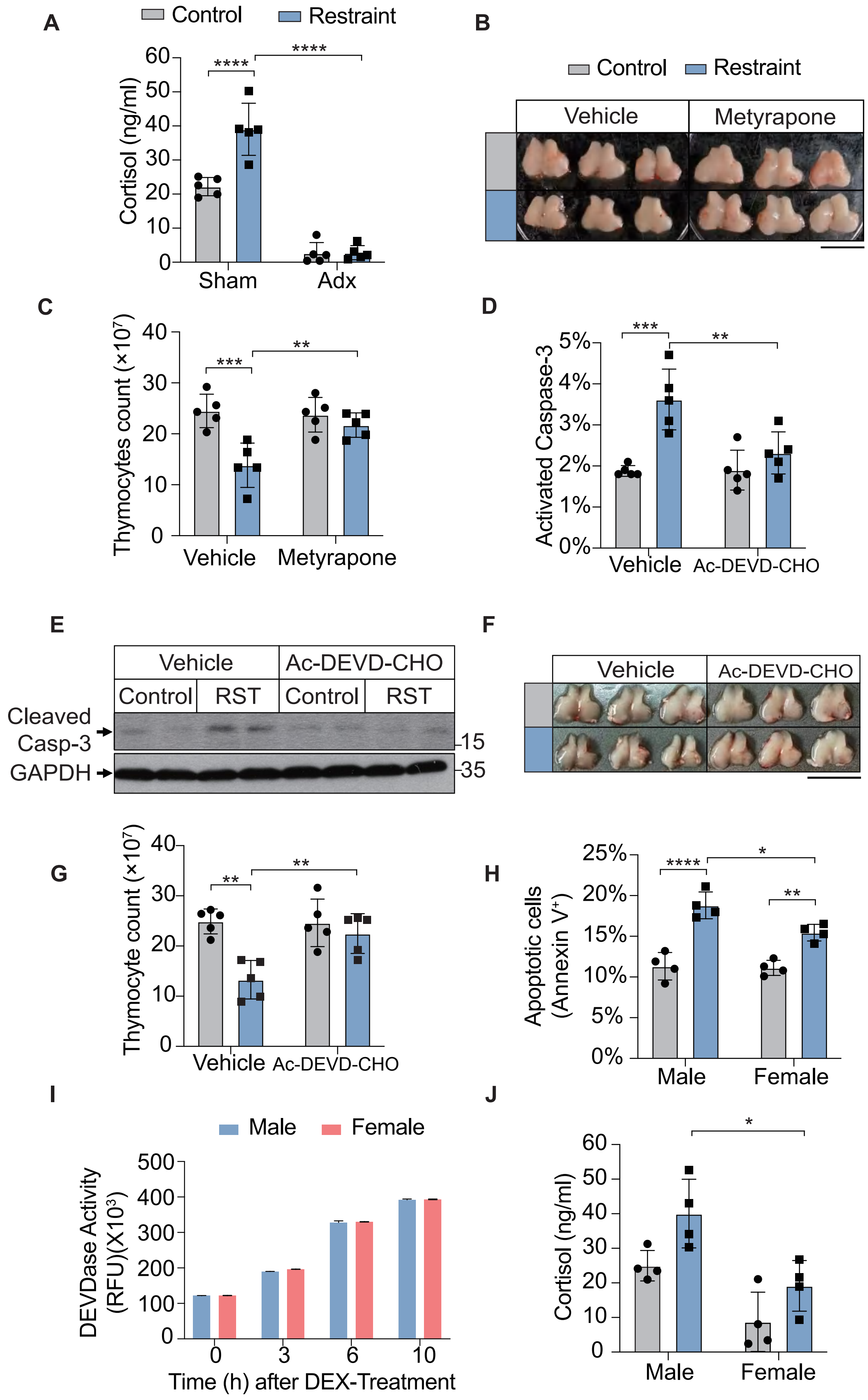

A
Control
Restraint
Cortisol (ng/ml)
60
50
40
30
20
10
0
****
****
Sham
Adx
B
Control
Restraint
Vehicle
Metyrapone
C
Thymocytes count (×10⁷)
40
30
20
10
0
***
**
Vehicle
Metyrapone
D
Activated Caspase-3
5%
4%
3%
2%
1%
0%
***
**
Vehicle
Ac-DEVD-CHO
E
Vehicle
Ac-DEVD-CHO
Control
RST
Control
RST
Cleaved Casp-3
GAPDH
15
35
F
Vehicle
Ac-DEVD-CHO
G
Thymocyte count (×10⁷)
40
30
20
10
0
**
**
Vehicle
Ac-DEVD-CHO
H
Apoptotic cells (Annexin V⁺)
25%
20%
15%
10%
5%
0%
****
*
**
Male
Female
I
Male
Female
DEVDase Activity (RFU)(X10³)
500
400
300
200
100
0
0
3
6
10
Time (h) after DEX-Treatment
J
Cortisol (ng/ml)
60
50
40
30
20
10
0
*
Male
Female

Fig. S1. Restraint-induced thymic atrophy depends on T cell apoptosis. (*A*) Serum cortisol levels in WT male mice subjected to sham surgery or adrenalectomy (Adx) under control or restraint stress conditions. n=5 mice per group. (*B*) Representative images of the thymus size in WT male mice treated with vehicle or Metyrapone under control (Control) or restraint stress (Restraint) conditions. Scale bar, 1cm. (*C*) Thymocyte counts in mice treated with vehicle or Metyrapone under control or restraint stress conditions. n=5 mice per group. (*D*) Proportions of thymocytes with activated caspase-3 in WT male mice treated with vehicle or the caspase inhibitor Ac-DEVD-CHO under control or restraint stress conditions. n=5 mice per group. (*E*) Immunoblot analysis of cleaved caspase-3 p17 (Cleaved Casp-3) and GAPDH in thymocytes from WT male mice treated with vehicle or Ac-DEVD-CHO under control or restraint stress (RST) conditions. Each lane represents a sample from an individual mouse. (*F*) Representative images of thymus size in mice treated with vehicle or Ac-DEVD-CHO under control (Control) or restraint stress (Restraint) conditions. Scale bar, 1cm. (*G*) Thymocyte counts in mice treated with vehicle or Ac-DEVD-CHO under control or restraint stress conditions. n=5 mice per group. (*H*) Proportions of apoptotic cells (Annexin V^+) in the thymus of male and female mice under control or restraint stress conditions. n=4 mice per group. (*I*) Caspase-3 activity (DEVDase Activity) in primary cultured thymocytes from male and female mice treated with 100 nM DEX at the indicated time points. (*J*) Serum cortisol levels in male and female mice under control or restraint stress conditions. n = 4 mice per group. For *A*, *C*, *D*, *G*, *H*, and *J*, data are presented as mean ± SD. Statistical analysis was performed using two-way analysis of variance (ANOVA) (*A*, *C*, *D*, *G*, *H*, and *J*,); *p<0.05, **p<0.01, ***p<0.001, ****p<0.0001.

Fig. S2

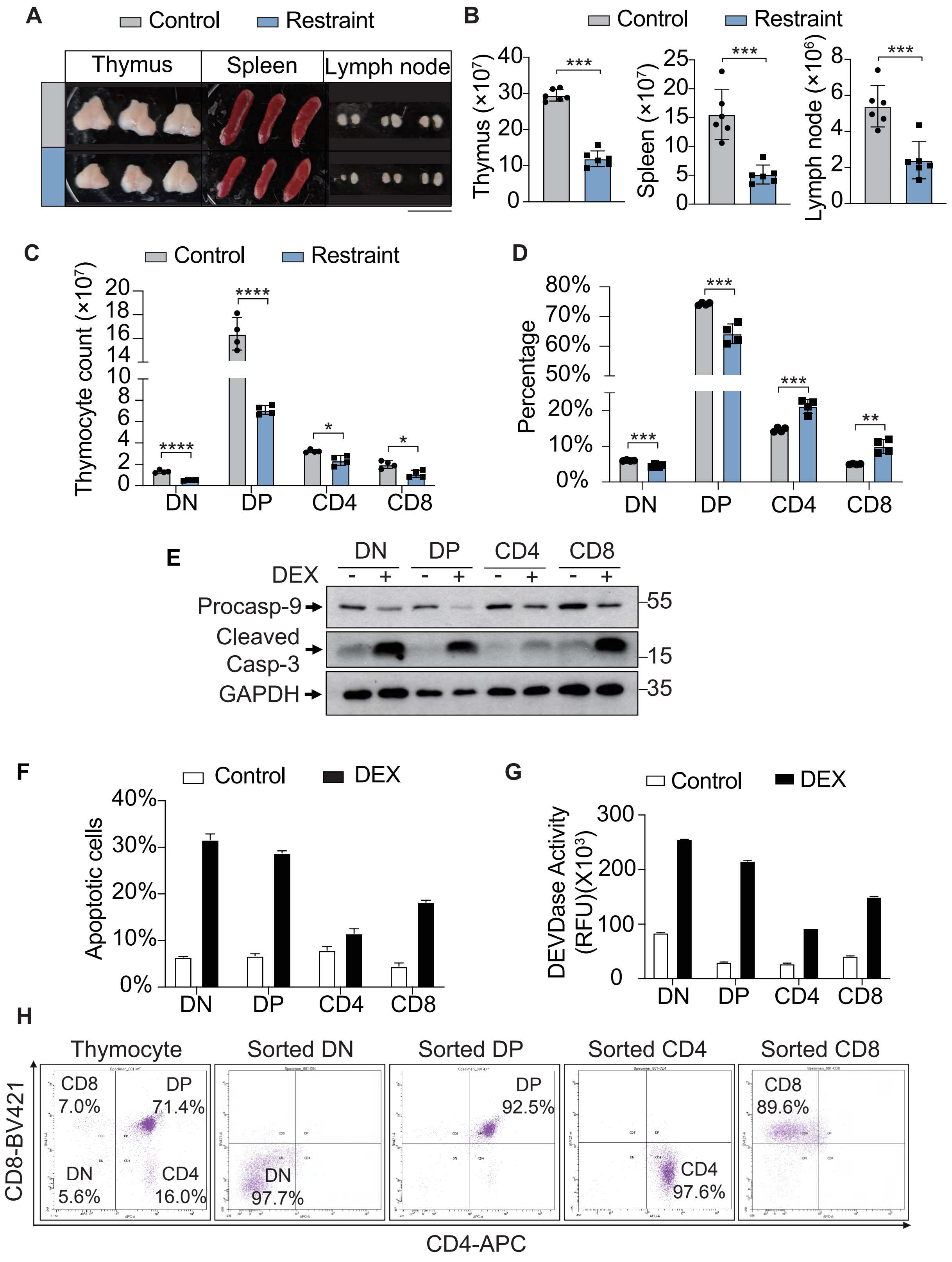
A
Control
Restraint
Thymus
Spleen
Lymph node
B
Control
Restraint
Thymus (×10^7)
Spleen (×10^7)
Lymph node (×10^6)
C
Control
Restraint
Thymocyte count (×10^7)
DN
DP
CD4
CD8
D
Percentage
DN
DP
CD4
CD8
E
DN
DP
CD4
CD8
DEX
Procasp-9
Cleaved Casp-3
GAPDH
55
15
35
F
Control
DEX
Apoptotic cells
DN
DP
CD4
CD8
G
Control
DEX
DEVDase Activity (RFU)(X10^3)
DN
DP
CD4
CD8
H
Thymocyte
Sorted DN
Sorted DP
Sorted CD4
Sorted CD8
CD8-BV421
CD4-APC
CD8 7.0%
DP 71.4%
DN 5.6%
CD4 16.0%
DN 97.7%
DP 92.5%
CD4 97.6%
CD8 89.6%

Fig. S2. Sensitivity of different T cell lineages to restraint stress and glucocorticoid. (*A*) Representative images showing the sizes of the thymus, spleen, and lymph nodes in male mice under control (Control) or restraint stress (Restraint) conditions. Scale bar, 1cm. (*B*) Quantification of cell numbers in thymus, spleen, and lymph nodes of male mice under control or restraint stress conditions. n=6 mice per group. (*C*) Cell numbers of T cell lineages in the thymus of male mice under control (Control) or restraint stress (Restraint) conditions. T cell lineages include $CD3^{+}CD4^{-}CD8^{-}$ double-negative (DN) T cells, $CD3^{+}CD4^{+}CD8^{+}$ double-positive (DP) T cells, $CD3^{+}CD4^{+}$ single-positive (CD4) T cells, and $CD3^{+}CD8^{+}$ (CD8) single-positive T cells. n=4 mice per group. (*D*) Proportions of T cell lineages in the thymus of male mice under control (Control) or restraint stress (Restraint) conditions. (*E*) Immunoblot analysis of procaspase-9 (Procasp-9), cleaved caspase-3 (Cleaved Casp-3), and GAPDH in different T cell lineages from the thymus treated with and without DEX (100 nM, 6 hours). (*F*) The proportions of apoptotic cells (Annexin V^{+}) among different T cell lineages in the thymus treated without (control) or with DEX (100 nM, 6 hours). (*G*) DEVDase Activity in different T cell lineages from the thymus treated with and without DEX (100 nM, 6 hours). (*H*) Sorted different T cell lineages by flow cytometry. The left panel shows the total input of thymic T cell populations. The four panels on the right show the sorted populations of DN, DP, CD4, and CD8 T cells. For *B*, *C*, *D*, *F*, and *G*, data are presented as mean ± SD. Statistical analysis was performed using two-way analysis of variance (ANOVA) (*C* and *D*) and two-tailed unpaired t-tests (*B*); *p<0.05, **p<0.01, ***p<0.001, ****p<0.0001.

Fig. S3

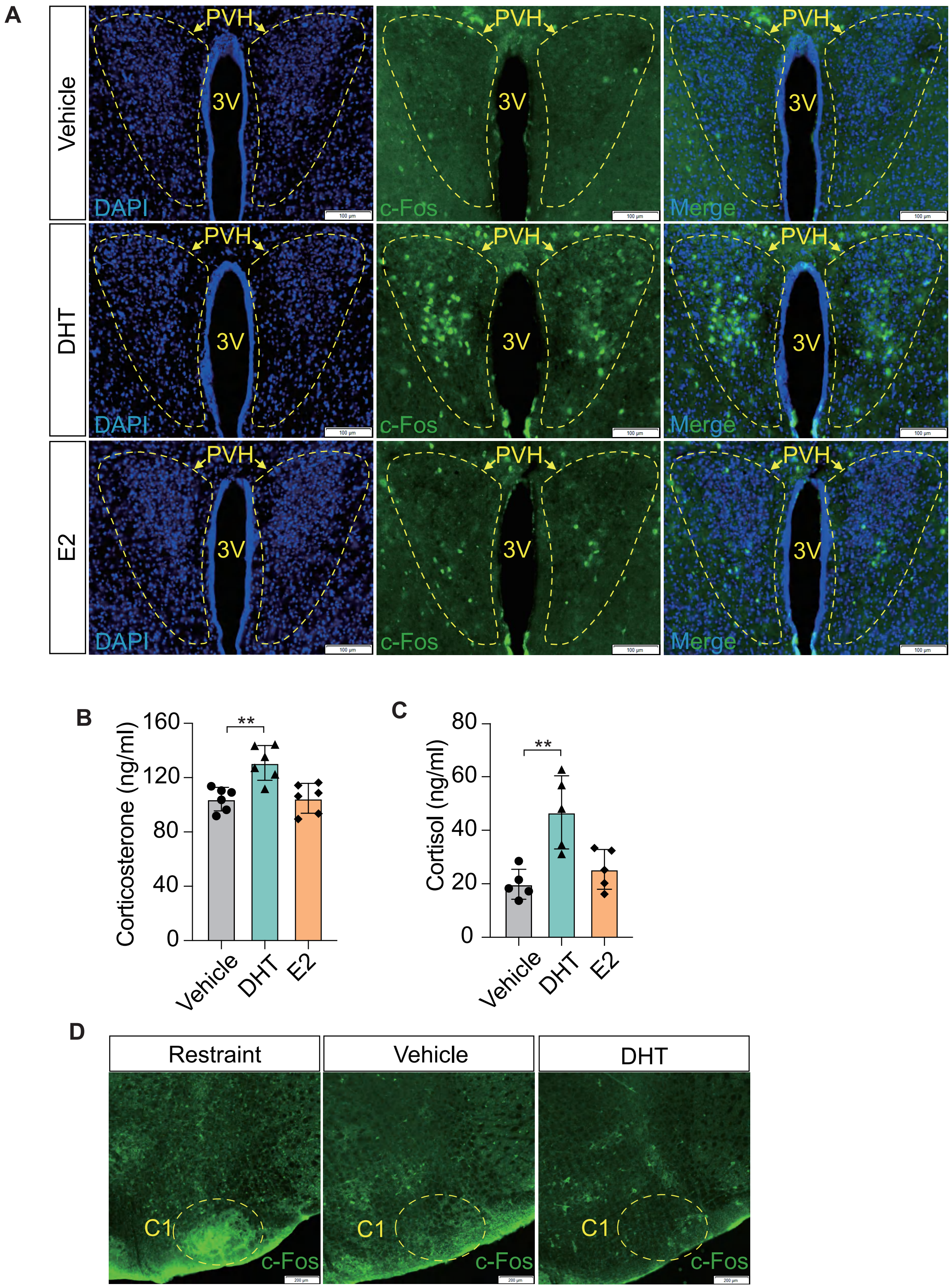
A
Vehicle
DHT
E2
PVH
3V
DAPI
c-Fos
Merge
B
Corticosterone (ng/ml)
160
120
80
40
0
**
Vehicle
DHT
E2
C
Cortisol (ng/ml)
80
60
40
20
0
**
Vehicle
DHT
E2
D
Restraint
Vehicle
DHT
C1
c-Fos

Fig. S3. DHT, rather than E2, activates the HPA axis. (*A*) Immunofluorescence staining of DAPI and c-Fos in the PVH from male mice treated with vehicle, DHT, or estradiol (E2). Scale bar, 100 μm. (*B*) Serum corticosterone levels in male mice treated with vehicle, DHT, or E2. n=6 mice per group. (*C*) Serum cortisol levels in male mice treated with vehicle, DHT, or E2. n=5 mice per group. (*D*) Immunofluorescence staining of c-Fos^{+} C1 neurons in male mice following restraint stress and in male mice treated with vehicle and DHT. Scale bar, 200 μm. For *B* and *C*, data are presented as mean ± SD. Statistical analysis was performed using one-way analysis of variance (ANOVA) (*B* and *C*); *p<0.05, **p<0.01, ***p<0.001.

Fig. S4

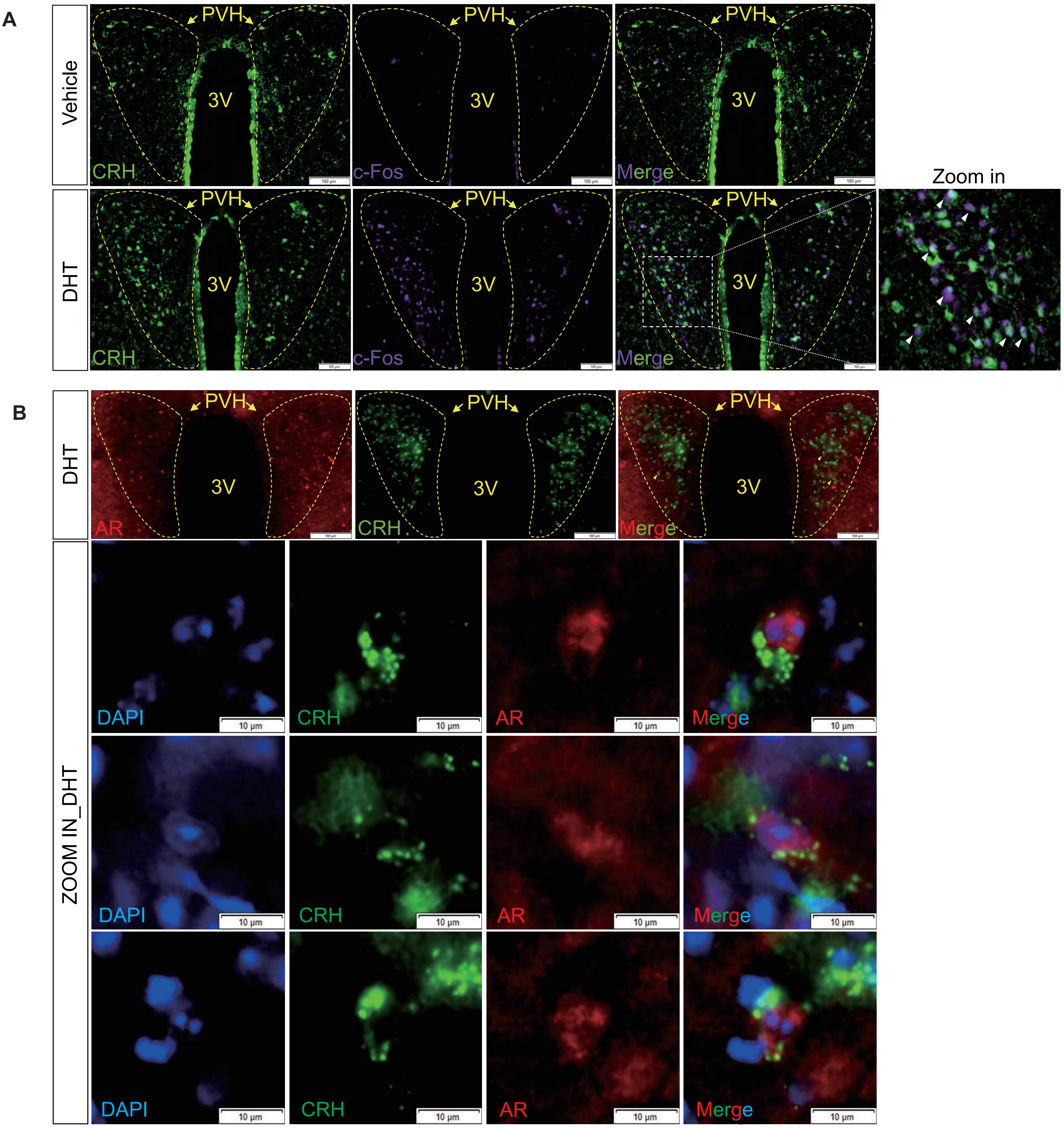
A
Vehicle
DHT
PVH
3V
CRH
c-Fos
Merge
Zoom in
B
DHT
ZOOM IN_DHT
AR
CRH
Merge
DAPI
10 μm

Fig. S4. Androgen receptor expression in CRH neurons. (*A*) Immunofluorescence staining of corticotropin-releasing hormone (CRH) and c-Fos in the PVH from WT male mice treated with vehicle or DHT. Scale bar, 100 μm. The enlarged image corresponds to the region outlined by the white dashed box. White arrows indicate the colocalization of CRH and c-Fos. (*B*) Immunofluorescence staining of androgen receptor (AR) and CRH in the PVH of WT male mice. Scale bar, 100 μm. Enlarged views of the area marked by the yellow arrows are shown below. Scale bar, 10 μm.

Fig. S5

A

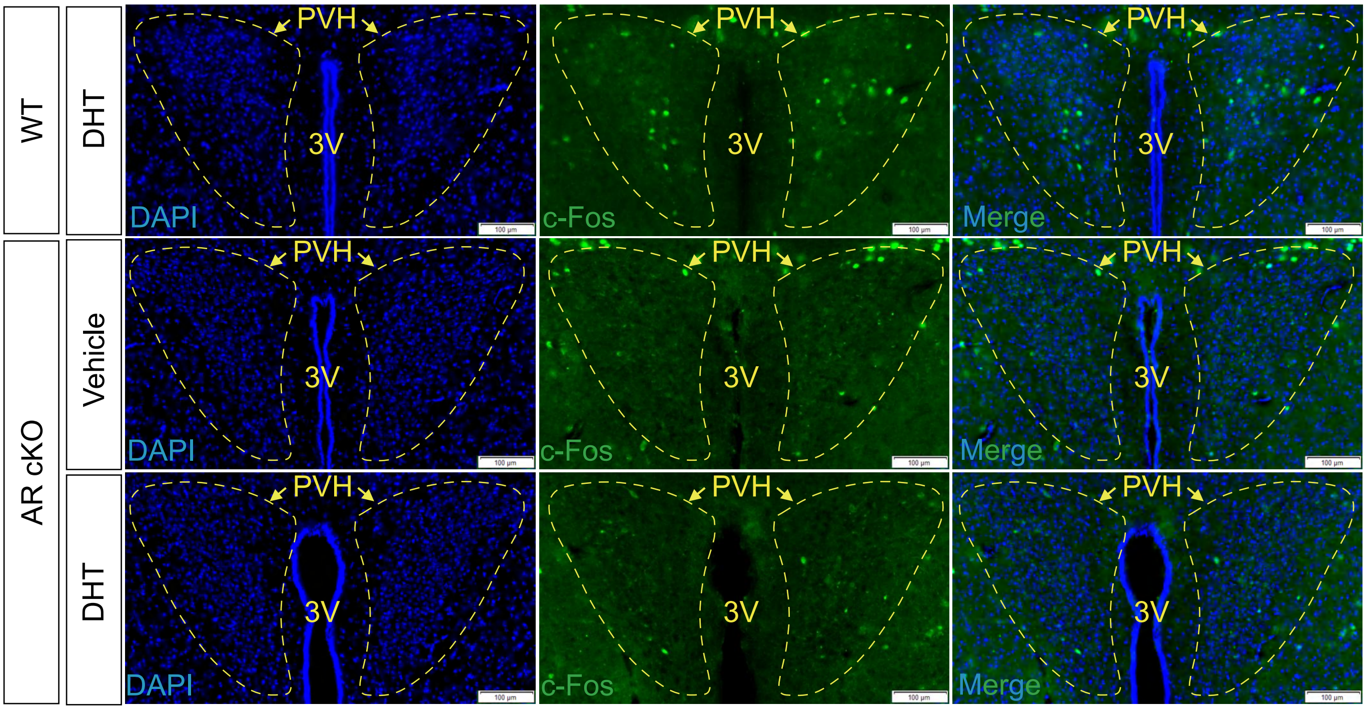
WT
AR cKO
DHT
Vehicle
DHT
PVH
3V
DAPI
c-Fos
Merge

B

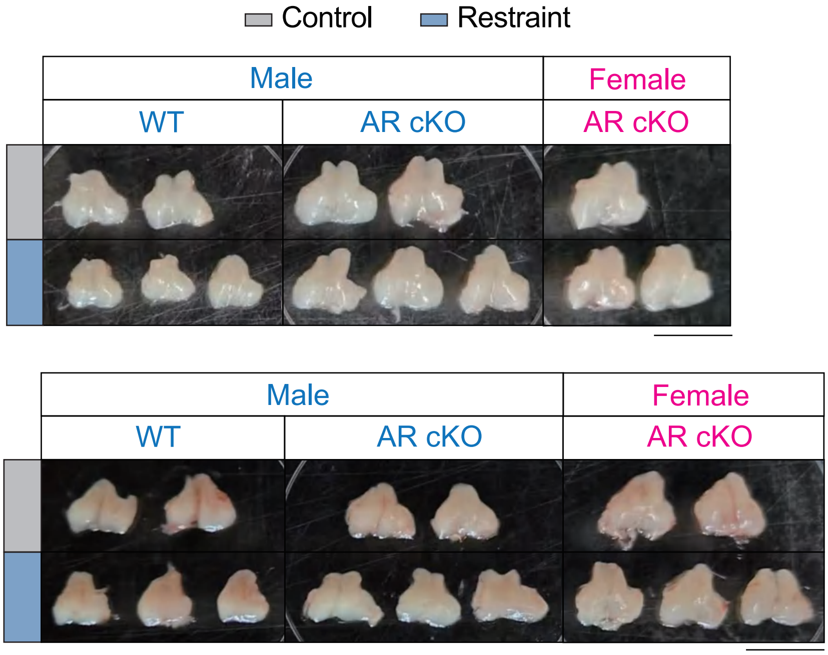
Control
Restraint
Male
Female
WT
AR cKO
AR cKO
Male
Female
WT
AR cKO
AR cKO

Fig. S5. AR cKO prevents DHT-induced activation in PVH neurons. (*A*) Immunofluorescence staining of DAPI and c-Fos in the PVH from wild-type (WT) and androgen receptor conditional knockout (AR cKO) male mice treated with vehicle or DHT. Scale bar, 100 μm. (*B*) Representative images of the thymus from male WT, male AR cKO, and female AR cKO mice under control (Control) or restraint stress (Restraint) conditions. Scale bar, 1cm.

Movie S1. 3D visualization of AR, c-Fos, and CRH in the PVH region of *Crh*$^{cre+/+}$; *Ai6*$^{+/+}$ male mice following DHT treatment.
